# Treatment of oligometastatic disease in colorectal cancer: from molecular biology to multimodal strategies

**DOI:** 10.3389/or.2026.1932297

**Published:** 2026-10-05

**Authors:** G. Arrichiello, A. Piccolo, F. Carlino, E. Franzese, A. Ventriglia, M. Annunziata, V. Patanè, M. De Siero, M. D. Bisceglia, M. Orditura

**Affiliations:** 1 Medical Oncology Unit, Azienda Ospedaliera di Rilievo Nazionale Sant’Anna e San Sebastiano Caserta, Casert, Italy; 2 Interventional Radiology Unit, Azienda Ospedaliera di Rilievo Nazionale Sant’Anna e San Sebastiano Caserta, Casert, Italy; 3 Pharmacy Unit, Azienda Ospedaliera di Rilievo Nazionale Sant’Anna e San Sebastiano Caserta, Casert, Italy

**Keywords:** ablation, colorectal cancer, oligometastatic, radiotherapy, surgery

## Abstract

The management of oligometastatic colorectal cancer (OMD-CRC) has undergone a profound evolution, transforming what was once a uniformly fatal diagnosis into a clinical scenario in which long-term survival and even cure are attainable for a carefully selected subset of patients. This review provides a comprehensive synthesis of integrated, multidisciplinary strategies for OMD-CRC, with particular emphasis on hepatic and pulmonary metastases. We examine the biological foundations that differentiate oligometastatic from polymetastatic disease, including molecular subtypes that inform prognosis and therapeutic responsiveness. Local treatment modalities are critically evaluated, ranging from surgical metastasectomy—the standard of care in adequately selected patients—to minimally invasive approaches such as thermal ablation and Stereotactic Body Radiotherapy (SBRT). Recent randomized trials and prospective studies have suggested possible non-inferiority of these alternatives to surgery in selected contexts, thereby broadening the spectrum of curative-intent interventions. Equally pivotal is the integration of systemic therapy. We discuss optimal timing (neoadjuvant, adjuvant, perioperative) and regimen selection for both resectable and initially unresectable disease. The role of conversion therapy, combining intensive chemotherapy with targeted agents guided by molecular determinants such as RAS/BRAF mutational status and tumor sidedness, is highlighted as a cornerstone in expanding surgical eligibility. Looking ahead, emerging technologies—including radiomics and liquid biopsy approaches such as circulating tumor DNA (ctDNA)—hold promise for refining patient selection, detecting minimal residual disease, and further individualizing treatment pathways. The convergence of advanced local therapies, precision-guided systemic regimens, and novel biomarkers delineates the new frontier in optimizing outcomes for patients with OMD-CRC.

## 1 Introduction

Globally, colorectal cancer (CRC) is the third most commonly diagnosed malignancy and the second leading cause of cancer-related mortality (1). Approximately half of all patients with CRC will eventually develop metastatic disease, with nearly 50% of these cases occurring after initial treatment for localized CRC (2, 3).

The overall 5-year relative survival rate for all stages of CRC is 65.0% (1). However, this figure declines sharply to 15.6% in stage IV disease, or metastatic CRC (mCRC). Despite this poor prognosis, survival outcomes for patients with mCRC have improved in recent years, largely due to advances in multidisciplinary management strategies that integrate systemic therapy with local treatment of both primary and metastatic lesion (4).

Within this spectrum, a distinct subgroup of patients with a limited metastatic burden may achieve prolonged survival. In contrast to widespread poly-metastatic disease (PMD), Hellman and Weichselbaum introduced the concept of “oligometastases,” describing a less aggressive disease state characterized by a restricted number of metastatic sites (5). Patients with oligometastatic disease (OMD) generally exhibit a more favorable prognosis and, in selected cases, may achieve cure when disease control is accomplished through a combination of local and systemic therapies (6).

Indeed, the median survival for patients with oligometastatic CRC is generally longer than for those with poly-metastatic CRC (38.2 versus 22.0 months; p < 0.01) (7).

Accordingly, precise definition and classification of oligometastatic CRC are critical for guiding optimal treatment strategies. While consensus exists on several aspects of OMD, standardized criteria and therapeutic approaches remain incompletely established and often vary across studies, medical disciplines, and clinical practice.

We produced this review aimed at the medical oncologist, to explore the management of oligometastatic CRC by defining the disease and outlining current therapeutic strategies. We underscore the integration of systemic therapy with local radical treatments, guided by molecular determinants and modern imaging. We also briefly explore potential future applications of novel technologies, such as liquid biopsy and radiomics, in the patient selection. For the sake of clarity and depth, the review focuses exclusively on the management of hepatic and pulmonary metastases, as other sites (peritoneum, brain, and bone) fall outside the synthesized scope of this narrative.

## 2 Methods

This narrative review synthesizes multidisciplinary management strategies for oligometastatic CRC, drawing on current guidelines, randomized trials, retrospective analyses, and meta-analyses identified through PubMed and Google Scholar searches (July 2025–February 2026) using terms including “colorectal cancer,” “oligometastases,” “local treatment,” “surgery,” “ablation,” “radiotherapy,” and “intra-arterial,” supplemented by relevant trials retrieved from ClinicalTrials.gov. Selection prioritized clinical relevance and publications from the last 10 years. Notably, most retrieved evidence derives from palliative or salvage settings, warranting caution in generalizing conclusions.

Ethics approval was not required given the narrative design; no new data were generated or analyzed. Inherent limitations include the absence of a systematic search protocol, lack of formal quality appraisal, and potential publication bias favoring studies with positive results.

## 3 Results

### 3.1 Oligometastatic disease: proposed classifications

The term “oligometastases” was first introduced in 1995 by Hellman and Weichselbaum to describe a clinical state characterized by a limited number of distant relapses amenable to local treatment (5). This concept was subsequently refined by Niibe et al., who proposed the term “oligo-recurrence” to define patients with a controlled primary tumor and one to five metachronous metastases, emphasizing that the status of the primary lesion represents the most critical prognostic determinant (8).

More recently, the European Society for Radiotherapy and Oncology (ESTRO) and the European Organisation for Research and Treatment of Cancer (EORTC) developed a comprehensive classification system delineating nine distinct states of oligometastatic disease (9). This framework differentiates between “genuine” OMD (no prior history of disseminated disease) and “induced” OMD (a history of widespread disease), with further subclassifications including synchronous, metachronous, oligorecurrence, and oligoprogression.

Nevertheless, the lack of standardized and universally accepted definitions continues to hinder the design and comparability of clinical trials. A multidisciplinary framework, analogous to that established for gastro-esophageal adenocarcinoma, would be highly desirable to reconcile classification and optimize therapeutic strategies (10).

Given that the lack of such definitions, works featured in this review enrolled patients with different characteristics–some with higher lesion counts. This makes it impossible to provide generalized recommendations; however, given the narrative nature of this paper the authors feel confident that it will nevertheless provide the reader with the tool to guide patient-informed choices in real life practice.

We acknowledge pre-existing pragmatic definitions, identifying OMD as 1–3 metastatic tumors per organ with a maximum size of less than 7 cm, or more stringent ones such as 1–5 tumors and a size limit of 5 cm (11).

However, we also believe that an operational definition of oligometastatic disease could apply to patients with disease amenable to radical treatment, either before or after successful systemic therapy and therefore we will reference this concept when addressing oligometastatic disease throughout the paper. It is important to notice that this is an operational clinical perspective rather than a universally accepted standard.

### 3.2 Biological foundations and molecular profile of oligometastatic CRC

Several efforts have been undertaken to refine the definition of oligometastatic colorectal cancer (OMD-CRC) through molecular characterization. At the time of reporting, clinical applicability of this method remains limited and should therefore be evaluated prospectively in a dedicated scenario. We also highlight that a key limitation to studying OMD is that, currently, identification of oligometastatic patients is done retrospectively and after a long observation of disease trajectory (11).

A framework has been proposed to classify colorectal liver metastases (CRLM) into three distinct molecular subtypes based on integrated mRNA and miRNA profiling (12).

These include: the *Immune* subtype (Subtype 2), associated with the most favorable survival and an oligometastatic pattern of recurrence characterized by few, limited lesions; the *Stromal* subtype (Subtype 3), linked to poor outcomes; and the *Canonical* subtype (Subtype 1), associated with intermediate prognosis.

An integrated model combining molecular subtypes with a clinical risk score (CRS) stratified patients into low-, intermediate-, and high-risk groups. The low-risk group, comprising patients with the immune subtype and a low CRS, representing approximately 22% of cases, achieved a 10-year overall survival of 94%, with all recurrences limited to one to three new lesions. We should emphasize that survival estimates derive from a selected retrospective cohort of patients undergoing resection of CRLM, rather than from prospective identification and longitudinal validation of an oligometastatic molecular state; therefore, caution is advised before applying these results broadly.

The immune subtype, which exhibits an oligometastatic phenotype, is characterized by robust immune activation, with strong infiltration of innate and adaptive immune cells (including B cells, NK cells, CD8^+^ T cells, and cytotoxic lymphocytes), independent of microsatellite instability (MSI). It is enriched for interferon-α and interferon-γ response signatures and uniquely associated with mutations in NRAS, CDK12, and EBF1. Mutations in ARID2 and other subtype-2-specific genes were also linked to enhanced cytotoxic immune responses. Compared with other subtypes, the immune subtype demonstrates a lower prevalence of VEGFA amplification.

By contrast, subtypes associated with worse outcomes and broader metastatic potential display distinct molecular features. The Stromal subtype is defined by high stromal infiltration, epithelial–mesenchymal transition (EMT), angiogenesis, and KRAS signaling, with exclusive somatic mutations in SMAD3 and frequent VEGFA amplification. The Canonical subtype is characterized by low immune and stromal signatures but enrichment for E2F/MYC signaling, altered DNA damage pathways, and cell cycle dysregulation; it harbors exclusive mutations in NOTCH1 and PIK3C2B, along with a high prevalence of VEGFA amplification. Notably, KRAS mutation, BRAF mutation, and MSI did not independently influence prognosis in this cohort.

Comparative genomic analyses of matched primary tumors and metastases identified atypical evolutionary trajectories involving KRAS and SMAD4. In lung-limited oligometastatic CRC, metastatic progression was associated with decreased tumor mutational burden, regression of KRAS and SMAD4 alterations, and site-specific genomic events such as ERBB2 mutations (13). Similar observations were subsequently reported in liver oligometastatic CRC, where loss of KRAS and SMAD4 alterations and increased granzyme B-positive lymphocyte infiltration characterized non-progressive disease, whereas acquisition of KRAS, PIK3CA, and SMAD4 alterations together with limited cytotoxic immune infiltration was associated with progression toward a polymetastatic phenotype (14). The clinical relevance of these evolutionary patterns was further supported by the observation that KRAS mutational regression from primary tumors to metastatic disease was strongly associated with oligometastatic status and favorable prognosis, whereas progressive KRAS trajectories were associated with poorer survival (15). Subsequent mechanistic analyses also suggested that immune-mediated clonal selection may contribute to these regressive trajectories, as regression of specific KRAS-mutated clones was associated with increased intratumoral granzyme B-positive lymphocyte density and enhanced CD3+/CD8+-dependent cytotoxicity against CRC cells carrying corresponding KRAS mutations (16). Overall, these findings support a model in which oligometastatic CRC may arise from the interaction between constrained genomic evolution and effective immune surveillance, thereby limiting metastatic diversification and systemic dissemination.

A separate study comparing OMD and poly-metastatic disease (PMD) in colon cancer further reinforced these findings (17). OMD was significantly more frequent in patients lacking KRAS mutations or specifically the BRAF p.V600E mutation. Moreover, OMD was associated with a higher prevalence of elevated tumor mutational burden (TMB), potentially facilitating enhanced immune surveillance and favoring a less aggressive metastatic phenotype. Conversely, loss of SMAD4 correlated with a poly-metastatic phenotype and poor prognosis. Mutations in BCL6 and FAT1 were also potentially associated with promoting aggressive, poly-metastatic disease.

### 3.3 Local treatment strategies: a modality-based approach

This section examines therapeutic modalities that have been investigated, either as standalone interventions or in combination, for the management of metastatic colorectal cancer lesions. Results presented should be interpreted with caution due to lack of standardized definition of OMD and dedicated clinical experiences, particularly when referring to intra-arterial therapies.

#### 3.3.1 Surgical metastasectomy: principles, indications, and organ-specific outcomes

##### 3.3.1.1 Liver

In the absence of randomized trials directly comparing surgical and non-surgical management, surgical resection has long remained the standard of care for patients with oligometastatic CRC.

In patients with metastatic CRC and a resected primary tumor, the impact of CRLM resection was evaluated through a SEER database analysis, which demonstrated that metastasectomy consistently improved cancer-specific survival in patients with liver metastases (HR = 0.72, 95% CI: 0.67–0.77, P < 0.001) (18). However, similar benefits were not observed in patients with lung-only or combined lung and liver metastases in this specific analysis. Although surgery is generally regarded as the only potentially curative option for CRLM, meticulous patient selection is essential (19). Based on resectability, CRLM can be classified as resectable, borderline/potentially resectable (following downsizing), or unresectable. Independent predictors of poor long-term outcomes, incorporated into the FONG score, include: node-positive primary tumor, disease-free interval <12 months, more than one hepatic metastasis, largest hepatic lesion >5 cm, and carcinoembryonic antigen (CEA) level >200 ng/mL (20).

Achieving complete tumor removal (R0 resection) while preserving adequate future liver remnant (FLR) volume is critical to prevent post-hepatectomy liver failure (21). The prognostic impact of margin status (R0 vs. R1) remains debated, with discordant evidence across studies (19). Parenchymal-sparing hepatectomy (PSH) has demonstrated comparable oncological outcomes to non-parenchymal-sparing approaches, while offering superior perioperative results and similar rates of positive margins (22). Additional surgical strategies include preoperative double-vein embolization (portal and hepatic) to induce FLR hypertrophy, two-stage hepatectomy for extensive bilateral disease, and ALPPS (Associating Liver Partition and Portal Vein Ligation for Staged Hepatectomy), a modified two-stage approach (19).

Multidisciplinary evaluation is essential to optimal selection among the different strategies.

Repeat local treatment for recurrent CRLM is feasible in patients with liver-only recurrence (23) Outcomes following a second hepatectomy show 5-year overall survival (OS) and progression-free survival (PFS) rates comparable to those after initial surgery. In carefully selected patients with low morbidity, survival may even surpass that of first-time resections, although outcomes generally decline with successive procedures (24).

Although outside the strict definition of oligometastatic disease, patients with liver-limited unresectable metastases may benefit from liver transplantation in addition to systemic chemotherapy (25). The TransMet randomized trial demonstrated that patients with BRAF wild-type metastatic CRC who achieved stable disease or response after ≥3 months of chemotherapy had markedly improved 5-year OS when allocated to transplantation (73.2% vs. 9.3% in the per-protocol analysis). Notably, 72% of patients in the transplant arm developed metastatic recurrence, predominantly in the lungs and lymph nodes; however, 46% underwent surgery or local ablation, achieving a secondary 5-year PFS rate of 36.1%.

##### 3.3.1.2 Lung

The therapeutic principles for managing pulmonary metastases from colorectal cancer (CRC) largely parallel those applied to colorectal liver metastases (CRLM). Surgical resection remains the standard of care in routine practice, typically yielding a 5-year overall survival (OS) of approximately 50%, with rates approaching 70% in carefully selected patient cohorts (26). The preferred surgical technique is generally wedge or segmental resection, with a recommended margin of 10–20 mm. Repeat pulmonary metastasectomy can also confer a survival benefit, particularly in patients with pulmonary oligometastases (27).

Prognosis is strongly influenced by disease-related factors, including the number and size of pulmonary lesions, the presence of extra-thoracic disease, and elevated preoperative carcinoembryonic antigen (CEA) levels (28–31).

In a retrospective cohort study conducted by Denz et al. at a German University Hospital, patients undergoing surgical resection of colorectal lung metastases demonstrated significantly superior outcomes compared with those receiving non-surgical management (32)). Among 418 patients, 2- and 5-year OS rates were 98.1% and 81.2% in the surgical group, compared with 67.9% and 28.8% in the non-surgical group, respectively. Similarly, a separate retrospective analysis of 1,647 patients—most with colorectal pulmonary metastases—reported 5-year OS and recurrence-free survival (RFS) rates of 62.0% and 29.6%, respectively (33).

Nonetheless, the survival benefit of surgical intervention has been questioned. A major meta-analysis of 16 randomized controlled trials (RCTs) found no significant survival advantage from diagnosing metastases 1–2 years earlier, suggesting that the increasing use of metastasectomy may not necessarily improve long-term outcomes (34) This conclusion was supported by a 2016 Cochrane review (35) and further reinforced by the PulMiCC randomized controlled trial, which confirmed that the benefit of lung metastasectomy may be less than previously believed (36).

Ultimately, surgery represents a mainstay option for the treatment of both liver and lung oligo-metastases from CRC; optimal patient selection and multidisciplinary discussion are functional to maximizing treatment potential.

#### 3.3.2 Image-guided ablative therapies: an alternative to surgery

##### 3.3.2.1 Liver

The use of percutaneous ablation for CRLMs is constrained by tumor size and anatomical location. In general, efficacy requires that the target lesion be ≤ 3 cm, with a tumor-free margin of at least 5–10 mm. Nevertheless, ablation of CRLMs up to 5 cm can be performed in carefully selected patients with appropriate planning and monitoring, yielding acceptable outcomes (37,38). Importantly, RAS-mutant tumors may necessitate wider margins to mitigate the risk of recurrence (39).

Several percutaneous ablation modalities are available. Radiofrequency ablation (RFA) remains the most widely used technique, while microwave ablation (MWA) and irreversible electroporation may be preferable for tumors adjacent to blood vessels or bile ducts, where the heat-sink effect compromises RFA efficacy (40).

Although the long-term effectiveness of ablation continues to be evaluated, historical data report survival rates of up to 50% in selected patients, albeit with variable local recurrence rates (41) The randomized phase II CLOCC trial compared systemic therapy alone versus systemic therapy plus RFA, demonstrating that the addition of RFA improved both progression-free survival (PFS) and overall survival (OS) (42).

Retrospective comparisons between surgery and other treatment modalities are inherently limited by selection bias. Surgical candidates typically represent a highly selected cohort with better performance status and fewer comorbidities, meaning observed survival advantages may reflect baseline patient fitness rather than true modality efficacy. Clinical factors driving treatment allocation—such as tumor size or proximity to critical structures—may themselves carry independent prognostic weight, further confounding comparisons. Outcomes are additionally influenced by institutional volume and the learning curve associated with each technique.

An umbrella review by Todeschini et al., synthesizing evidence from eleven meta-analyses, found that RFA was associated with significantly lower perioperative complication rates compared with surgery, but at the expense of higher intrahepatic and marginal recurrence (43). In matched cohort analyses designed to reduce selection bias, overall survival did not differ significantly between ablation and surgery, although disease-free survival continued to favor surgical resection.

The recently published COLLISION randomized trial (44)) enrolled patients with ≤10 small CRLMs (≤3 cm) and no extrahepatic disease. The study was terminated early after meeting predefined interim stopping rules, demonstrating non-inferiority of thermal ablation compared with surgery for overall survival (HR 1.05; p = 0.83). Ablation also achieved non-inferior local control and a superior safety profile. However, interpretation requires caution, since the premature halt limits definitive conclusions regarding non-inferiority, particularly in intermediate- and high-burden subgroups, which were underrepresented. Moreover, the trial was conducted in high-volume centers with highly experienced operators, restricting generalizability to smaller institutions.

Similar findings were reported in the MAVERRIC prospective trial, which compared stereotactic MWA with hepatic resection in patients with ≤5 CRLMs ≤30 mm, using control group data from a nationwide Swedish database. This study also supported non-inferiority of ablation relative to surgery (45).

Several ongoing trials aim to clarify unresolved issues. The NEW-COMET trial (NCT05129787) is evaluating 12-month local tumor progression after thermal ablation vs. surgical resection. The HELARC trial (NCT02886104) is comparing simultaneous resection of the primary tumor and liver metastases with staged resection of the primary tumor plus percutaneous thermal ablation of liver metastases. COLLISION-XL (NCT04081168) will assess ablation vs. stereotactic body radiotherapy for larger lesions.

A concise summary of relevant evidence regarding local approaches to CRLM is summarized in Table 1.

**TABLE 1:** Summary of relevant evidence regarding image-guided treatment of colorectal cancer liver metastases.

Study (Year)	Study type	Treatment	Population	Key outcome
van der Lei (2025)	Prospective phase 3 study	Thermal ablation vs. Surgery	300 pts (≤10 CRLM, ≤3 cm)	Non-inferior OS (HR 1.05) and superior safety profile
Tinguely (2023)	Prospective cohort trial	SMWA vs. Liver resection	98 pts (1–5 CRLM, ≤30 mm)	Similar 3-year OS (78% vs. 76%); significantly fewer complications with SMWA
Ruers (2017)	Prospective randomized phase 2 trial	Systemic + RFA vs. Systemic alone	119 pts (unresectable CRLM)	Improved mOS (45.6 vs. 40.5 months; p = 0.01) with added RFA
Schullian (2020)	Retospective study	SRFA	64 pts (recurrent CRLM)	5-year OS of 34.8%; high technical efficacy (97.7%)
Dijkstra (2023)	Retospective study	RFA & MWA	280 pts (small vs. intermediate lesions)	Superior local control for lesions <3 cm

CRLM, colorectal cancer liver metastases; mOS: median overall survival; RFA: radiofrequency ablation; SMWA: stereotactic microwave ablation.

##### 3.3.2.2 Lung

Percutaneous ablation represents a therapeutic option for patients with a limited number of pulmonary metastases from colorectal cancer, particularly in cases where surgery is contraindicated or when ablation is employed in conjunction with surgical resection (46).

A recent review summarizing data from 12 studies reported local control rates ranging from 62% to 91%, with median overall survival (OS) varying between 33 and 68 months (47)). Local control is strongly influenced by tumor size and the achievement of adequate margins, which may differ according to the ablation technique utilized (48).

In comparative analyses of ablation modalities, a retrospective study demonstrated that microwave ablation (MWA) achieved superior local control and greater tumor volume reduction compared with radiofrequency ablation (RFA); however, these advantages did not translate into differences in OS or time to progression (49).

Direct comparison between ablation and surgical metastasectomy remains challenging due to inherent patient selection bias, and to date, no prospective randomized trial has been conducted to definitively establish the relative efficacy of these approaches.

In conclusion, both for liver and lung metastases from CRC, ablation techniques provide a safer and potentially equally effective alternative to surgery in adequately selected patients.

#### 3.3.3 Stereotactic body radiotherapy (SBRT/SRS): Non-invasive ablation

##### 3.3.3.1 Liver

Stereotactic Body Radiation Therapy (SBRT) has been investigated as a treatment modality for CRLMs. A systematic review including 656 patients with colorectal liver oligometastases reported 2-year local control and OS rates of 59.3% and 56.5%, respectively, with limited severe liver toxicity (50). Notably, local control does not appear to correlate with tumor size, whereas radiation dose plays a critical role in determining outcomes (51).

The SABR-COMET randomized phase II trial, which enrolled a small cohort of colorectal cancer patients (18.2%), demonstrated that the addition of SBRT to standard care for oligo-recurrence improved both OS and progression-free survival (PFS) (52) Conversely, a pooled retrospective analysis conducted by the German Society for Radiation Oncology, encompassing 228 CRC patients, revealed that liver metastases from CRC exhibited significantly lower 1-year local control rates (67%) compared with metastases from breast cancer (91%) or non-small cell lung cancer (NSCLC) (88%) (53).

Although no randomized controlled trials have directly compared SBRT with other local treatment modalities, SBRT offers distinct advantages over ablation. Unlike ablation, SBRT is less constrained by tumor location, making it particularly suitable for lesions in anatomically challenging sites or adjacent to major blood vessels, where the “heat-sink” effect may compromise ablation efficacy (54, 55). Furthermore, SBRT may provide superior local control for larger tumors (>2–3 cm) compared with ablation (54, 56).

An innovative combined approach was evaluated in a retrospective study by Li et al., in which surgery was complemented by planned SBRT for residual CRLMs deemed unresectable or unablatable. This strategy was shown to be safe and effective, achieving a median OS of 45.8 months, with local failure rates correlating with overall tumor burden and grade 3 toxicities reported in 4.3% of patients (57).

As evidenced so far, the selection of the optimal local treatment modality for CRC liver oligo-metastases depends on a synthesis of patient fitness, anatomical constraints, and tumor biology. Surgical metastasectomy remains the standard of care in appropriately selected patients with minimal comorbidities, provided an R0 resection is achievable while maintaining sufficient residual organ function. For patients who are surgically unfit or present with recurrent disease, the choice between alternative treatment modalities should be guided by a multidisciplinary assessment of the patient’s overall health and the specific technical feasibility of achieving complete local eradication.

##### 3.3.3.2 Lung

SBRT has also demonstrated efficacy in the treatment of pulmonary metastases from colorectal cancer, with most evidence derived from retrospective studies. In patients with unresectable pulmonary oligometastases, one study reported 2-year and 5-year local control rates of 83% and 77%, respectively, with corresponding overall survival (OS) rates of 69% and 36%. Importantly, outcomes were superior in patients receiving higher biologically effective doses (BED) (58). In the largest retrospective series to date, the LaIT-SABR study, which included 1,033 pulmonary oligometastases, 2-year local progression-free survival (PFS) was significantly higher at 94% when BED was ≥125 Gy compared with lower doses. This study also identified lesion size >20 mm and the presence of 4–5 simultaneous oligometastases as predictors of polymetastatic progression.

A network meta-analysis by Chierici et al. demonstrated that SBRT is particularly effective when integrated into multimodal treatment strategies for pulmonary metastases. Across 11 studies, the combination of surgery, SBRT, and chemotherapy emerged as the most effective overall approach (59).

To summarize, SBRT could represent an alternative to both surgery and ablation in patients unsuitable for either due to disease or patient characteristics, offering acceptable toxicity and adequate efficacy in challenging situations.

#### 3.3.4 Intra-arterial locoregional therapies for liver-dominant disease

Intra-arterial therapies hold promise for the treatment of liver-prevalent disease, but most of the evidence accumulated was produced in the palliative setting, so that immediate translation into the oligometastatic scenario requires caution.

Conventional transarterial chemoembolization (cTACE) and irinotecan-loaded drug-eluting beads TACE (DEBIRI-TACE) have been investigated for the management of CRLMs. In the salvage setting, a large retrospective study of 564 patients reported a median survival of 14.3 months following cTACE (60). The same group subsequently demonstrated improved outcomes when cTACE was administered as neoadjuvant therapy prior to ablation of CRLMs, compared with its use as a stand-alone treatment in a different retrospective analysis (61). Prospective clinical trials evaluating DEBIRI-TACE in patients previously treated with systemic chemotherapy reported encouraging median progression-free survival (PFS) and median overall survival (OS) (62). In a direct comparison with systemic FOLFIRI, DEBIRI-TACE achieved a median OS of 22 months versus 15 months in the FOLFIRI group. Furthermore, combining DEBIRI-TACE with systemic chemotherapy in patients with unresectable CRLM improved overall response rates, hepatic PFS, and overall PFS, with some patients successfully downsized to resection in a randomized controlled trial (63).

More recently, a prospectively observational study, the CIREL trial, evaluated DEBIRI-TACE as salvage or post-inductive therapy in CRLM (64). In the salvage group, median OS was 9.9 months and median PFS was 3.8 months. In contrast, patients receiving irinotecan-TACE as post-inductive/consolidation therapy achieved more favorable outcomes, with median OS of 19.1 months, median PFS of 6.0 months, and median hepatic PFS of 8.7 months.

For patients with liver-limited metastases ineligible for further chemotherapy, transarterial radioembolization (TARE) with Y-90 resin microspheres has also been explored. A large analyses involving >1,000 patients across three randomized trials found no OS benefit when TARE was added to first-line systemic therapy (65). Similarly, a phase III trial of TARE with resin microspheres did not demonstrate a benefit in overall PFS, though it did show a significantly improved ‘liver-specific-PFS’ (66), suggesting that TARE may be most appropriate for patients with liver-limited or liver-predominant disease. The EPOCH phase III prospective trial further evaluated second-line chemotherapy alone versus chemotherapy combined with transarterial Y-90 glass microspheres in 428 patients with liver-dominant or liver-only disease. Combination therapy significantly improved PFS, with objective response rates (ORRs) of 34.0% versus 21.1% for chemotherapy alone. Subgroup analysis indicated that the PFS benefit was most pronounced in patients with fewer than three lesions, resected primary tumors, lower tumor burden, left-sided primaries, and KRAS mutations.

Hepatic arterial infusion chemotherapy (HAIC), typically involving floxuridine (FUDR) or oxaliplatin delivered via implanted ports, has also shown promise. In heavily pretreated patients within prospectively maintained registries, HAIC with oxaliplatin achieved an ORR of 62% and a complete pathological response rate more than nine-fold higher than systemic therapy (67, 68). The phase II OPTILIV study combined HAIC (irinotecan–oxaliplatin–5-FU) with intravenous cetuximab in 64 pretreated RAS wild-type patients with extensive CRLM. The study met its primary endpoint, enabling 29.7% of patients to undergo curative-intent (R0–R1) hepatectomy (69). A recent meta-analysis confirmed survival and response benefits for HAIC, both as palliative therapy in unresectable disease and as adjuvant therapy following curative resection (70).

In conclusion, intra-arterial therapies could potentially complement systemic chemotherapy as well as other locoregional treatment options for patients with CRLM.

Table 2 summarizes the most relevant evidence on intra-arterial therapies for colorectal liver metastases.

**TABLE 2:** Intra-arterial locoregional therapies for liver-dominant disease.

Setting	Study (Year)	Study type	Treatment	Population	Key outcome
Salvage	Arnold (2025)	Prospective observational study	DEBIRI-TACE	Heavily pretreated patients	mOS 9.9 months and median PFS of 3.8 months in the salvage cohort
Conversion/Consolidation	Arnold (2025)	Prospective observational study	DEBIRI-TACE	Post-inductive/consolidation	mOS 19.1 months and median PFS of 6.0 months
	Lévi (2016)	Prospective phase 2 study	HAI triplet + cetuximab	Unresectable KRAS-wt	29.7% achieved R0/R1 resection in previously treated patients
	Martin (2015)	Randomized phase 2 study	DEBIRI + systemic	Chemo-naive unresectable	Higher response rate (95% vs. 70%) at 4 months compared to systemic alone

mOS, median overall survival; PFS, progression-free survival; TTLP, time to liver progression; ORR, objective response rate; HAI, hepatic arterial infusion; DEBIRI-TACE, irinotecan-eluting bead chemoembolization; TACE, trans-arterial chemoembolization.

### 3.4 Integrating local and systemic therapies

Systemic therapy remains the cornerstone of treatment for mCRC, and its appropriate integration with local modalities is critical for the optimal management of oligometastatic disease. This section will examine the role of systemic chemotherapy in conjunction with locoregional strategies, both as adjuvant and as neoadjuvant/conversion therapy, and will further address the contribution of targeted agents within this therapeutic context. When addressing this topic readers should keep in mind that, historically, clinical trials in advanced settings have failed to clearly identify, stratify, or report on OMD. Standard trials often conflate OMD with a heterogeneously defined “low-burden disease,” which is frequently based strictly on the number of involved organs rather than actual tumor volume or size limits. This lack of precise reporting makes it difficult to extract reliable, high-quality evidence specifically applicable to OMD patients.

#### 3.4.1 Upfront resectable: perioperative, neoadjuvant, and adjuvant approaches

Most of the evidence regarding the positioning of chemotherapy in the management of OMD pertains to liver metastases. A 2025 meta-analysis by Tatsuta et al., focusing on studies published between 2019 and 2023, evaluated the impact of adjuvant chemotherapy (AC) following curative resection of CRC metastases (71). This analysis, which included 1,580 patients across seven studies (two randomized clinical trials and five retrospective trials), demonstrated that AC significantly improved disease-free survival (DFS). Improvement in OS, however, showed only a positive trend and did not reach statistical significance. Subgroup analysis revealed that the benefit was most pronounced in patients with liver metastases, where AC significantly improved DFS and suggested a trend toward better OS. Conversely, for lung metastases, AC did not confer any OS benefit.

A comprehensive 2025 review by Peixoto et al. corroborated these findings, noting that while AC after resection of colorectal liver metastases (CRLM) improves DFS, evidence for an OS benefit remains limited, likely due to the impact of subsequent treatments for recurrence, such as repeat hepatectomy (72). The authors proposed stratifying management into clinical scenarios: patients with metachronous metastases who have already received oxaliplatin-based AC are unlikely to benefit from additional AC and should be considered for surveillance, whereas patients with synchronous disease—considered more aggressive—are often recommended for perioperative or adjuvant chemotherapy.

Recommendations for perioperative chemotherapy in patients with upfront resectable liver metastases are largely based on the EORTC 40983 trial (73). Patients assigned to perioperative FOLFOX achieved longer 3-year PFS with a 7.3% absolute increase, although no OS benefit was observed. Bernardi et al. later suggested that any survival advantage may have been diluted by salvage chemotherapy in the surgery-alone arm (74) They also highlighted potential benefits of perioperative chemotherapy, including facilitation of parenchymal-sparing surgery and eradication of micrometastatic disease, while minimizing drawbacks such as chemotherapy-induced liver injury or vanishing lesions, thanks to advances in surgical and imaging techniques. The New EPOC trial, a direct extension of EORTC 40983, failed to demonstrate survival improvement with the addition of cetuximab to chemotherapy in KRAS exon 2 wild-type patients with resectable CRLM (75). Notably, survival was shorter in the experimental arm, possibly due to induction of more aggressive disease, as suggested by shorter post-progression survival and higher rates of multi-site progression.

More recently, a retrospective observational analysis of two Swedish national registries including 2,071 patients with synchronous CRLM reported a protective effect of adjuvant therapy (HR 0.80, 95% CI 0.69–0.94), whereas neoadjuvant therapy compared with upfront surgery did not influence OS (HR 1.04, 95% CI 0.86–1.26) (76).

#### 3.4.2 Upfront unresectable disease: chemotherapy, biologics, and immunotherapy

In accordance with the “operational” definition of OMD addressed earlier, we thought to be adequate to evaluate treatment opportunities in patients with upfront unresectable disease who achieve induced-OMD status because of systemic therapy. Lack of dedicated studies, however, prevents us from recommending a specific treatment regimen or duration of therapy in this clinical scenario.

For patients with upfront unresectable disease, current guidelines recommend the use of optimal conversion therapy (6). A 2020 systematic review by Bolhuis et al. investigated systemic therapy in patients with colorectal cancer liver-only metastases (CRLM) (87)). The authors concluded that, due to substantial inconsistencies across clinical trials, no definitive systemic regimen can be identified from the available data. Many studies were not specifically designed for CRLM but rather for the broader metastatic CRC population, with CRLM patients analyzed retrospectively as small subgroups. Moreover, the evolving understanding of prognostic and predictive biomarkers—such as RAS/BRAF mutation status and primary tumor sidedness—renders older trial data difficult to interpret in the modern context.

Prospective trials have evaluated bevacizumab-containing regimens in patients with initially unresectable CRLM in liver limited disease. The OLIVIA trial compared intensive triplet chemotherapy (FOLFOXIRI) plus bevacizumab with standard doublet (FOLFOX) plus bevacizumab, demonstrating significantly higher R0 resection rates (54% vs. 31%) with the triplet regimen (77). The BECOME trial, conducted in an Asian population, showed that adding bevacizumab to FOLFOX increased the R0 resection rate from 6% to 22% and improved median OS (25.7 vs. 20.5 months) (78). Trials using anti-EGFR agents in RAS wild-type patients also yielded notable results. The CELIM study reported R0 resection rates of 38% for FOLFOX-cetuximab and 30% for FOLFIRI-cetuximab (79). The ATOM trial directly compared FOLFOX-cetuximab with FOLFOX-bevacizumab, finding higher tumor response rates in the cetuximab arm (85% vs. 68%) but numerically lower resection rates with antiEGFR, challenging the assumption that higher response rates necessarily translate into improved surgical outcomes (80). Importantly, this trial included both left- and right-sided primaries, which must be considered when interpreting results.

Retrospective subgroup analyses of larger mCRC trials provide further insights into CRLM management. Unless otherwise specified, we will be referring to trials conducted in previously untreated patients. In the TRIBE and TRIBE-2 trials, triplet versus doublet chemotherapy, both combined with bevacizumab, yielded similar R0 resection rates in CRLM subgroups (81, 82). The HORIZON III and MAVERICC trials, both using FOLFOX-bevacizumab, reported markedly different resection rates (14% vs. 39%), underscoring the difficulty of cross-trial comparisons (83, 84). The pooled R0 resection rate for doublet-bevacizumab regimens in subgroup analyses was 19% (87). For anti-EGFR therapies, subgroup analyses of CRYSTAL, FIRE-3, and PEAK trials provided data for RAS wild-type patients. In FIRE-3, the CRLM subgroup achieved an R0 resection rate of 22% with FOLFIRI-cetuximab (85). The weighted pooled mean R0 resection rate for doublet chemotherapy plus anti-EGFR therapy across broader trials was 18%, substantially lower than the 34% observed in prospective CRLM-specific trials (86). The PARADIGM trial prospectively compared panitumumab versus bevacizumab added to standard first-line chemotherapy in RAS wild-type, left-sided mCRC, reporting higher response rates (80.2% vs. 68.6%) and higher curative (R0) resection rates (18.3% vs. 11.6%) with panitumumab (113). These findings were reinforced by ctDNA-based hyper-selection strategies to exclude patients with EGFR-resistance mechanisms (87).

Treatment intensification with FOLFOXIRI compared to FOLFOX plus panitumumab did not confer advantages in early tumor shrinkage (57% vs. 58%, P = 0.878), depth of response (median 48% vs. 47%, P = 0.845), or R0 resection rates (25% vs. 29%, P = 0.317), as demonstrated in the TRIPLETE trial (88). Similar conclusions were reached in the UNICANCER PRODIGE-14 trial, which failed to show increased R0/R1 resection rates in patients with unresectable liver-only CRLM treated with intensified regimens stratified by RAS status (89).

Initial results from the CAIRO5 trial were recently reported, with surgical perspectives provided by Meskauskas and Gelli (90). The trial enrolled patients with initially unresectable CRLM and stratified them by primary tumor sidedness and molecular profile. For patients with right-sided or RAS/BRAF mutated tumors, triplet chemotherapy (FOLFOXIRI) plus bevacizumab significantly improved PFS and increased complete (R0/R1) resection rates from 37% to 51%, compared with doublet chemotherapy plus bevacizumab. For patients with left-sided, non-mutated tumors, doublet chemotherapy plus panitumumab did not improve PFS or resection rates compared with doublet chemotherapy plus bevacizumab, although panitumumab yielded higher response rates and greater toxicity. Importantly, achieving secondary resectability conferred a significant survival benefit across all patient groups.

dMMR/MSI-H CRC patients represent a distinct subgroup of patients in which immune checkpoint inhibitors (ICI) offer a higher chance of prolonged disease control compared to standard cytotoxic chemotherapy (6). A recent retrospective observational analysis suggests high complete pathological response rate in patients with mCRC undergoing curative surgery after response to treatment ICIs, which could also potentially question the role of either surgery or ablation in patients with optimal clinical response (91). However, no dedicated prospective trial has been reported to date and evidence is insufficient to recommend a “conservative” approach or to further select dMMR/MSI-H patients for local treatment.

In Figure 1 we propose a practical framework to guide patient selection and treatment allocation for CRLM according to most relevant variables (e.g., tumor biology, surgical resectability, disease burden), once again highlighting the value of multidisciplinary team discussion.

**FIGURE 1:**
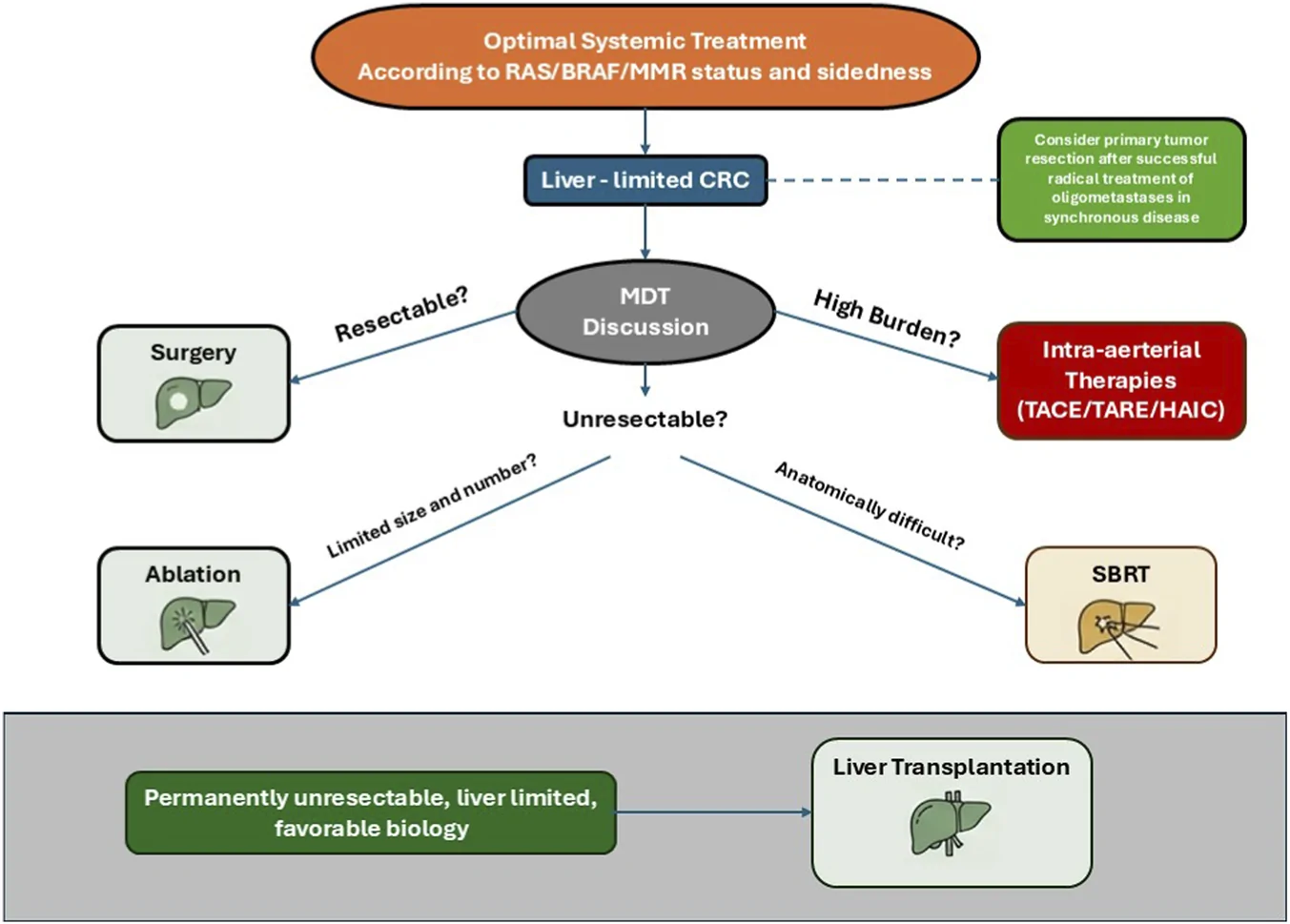
Patient-selection and treatment allocation framework for colorectal cancer liver metastases. CRC, colorectal cancer; HAIC, hepatic arterial infusion chemotherapy; MDT, multi-disciplinary team; SBRT, stereotactic body radiotherapy; TACE, trans-arterial chemoembolization; TARE, trans-arterial radioembolization.

### 3.5 Emerging techniques for patient selection and risk stratification

Two rapidly evolving fields are reshaping the management of OMD-CRC: radiomics and liquid biopsy. Radiomics extracts high-dimensional features from conventional imaging to decode tumor phenotypes, while liquid biopsy—primarily through circulating tumor DNA (ctDNA) analysis—enables detection of minimal residual disease and treatment monitoring. Together, they enhance patient stratification and support more precise therapeutic decision-making. Neither, however, has yet reached routine clinical implementation.

#### 3.5.1 Radiomics

Radiomics is an emerging discipline that harnesses advanced computational methods to convert standard medical images obtained from modalities such as MRI, CT, ultrasound, and PET into high-dimensional quantitative data. By extracting and analyzing features related to shape, intensity, volume, and texture, radiomics provides detailed insights into the tumor microenvironment and phenotype, with potential applications in cancer diagnosis, detection, prognostic assessment, prediction of treatment response, and monitoring of disease progression (92).

Texture analysis of apparently disease-free liver parenchyma on CT scans has been shown to distinguish patients who either harbor or are at risk of developing liver metastases from those who do not (93). Moreover, radiomics models derived from CT or MRI can non-invasively differentiate histopathological growth patterns (HGPs) of CRLMs, such as the desmoplastic and replacement types (94, 95). This distinction is clinically relevant, as HGPs are strongly associated with prognosis and therapeutic response, particularly to chemotherapy and anti-angiogenic agents (96) Radiomics has also been applied to evaluate and predict the response of CRLMs to systemic therapy. Features such as entropy, uniformity, and variance have been correlated with treatment efficacy and survival outcomes, underscoring the potential of radiomics as a predictive biomarker in this setting (97). A major hurdle in CRC radiomics is the lack of feature reproducibility across different imaging environments. Variations in scanner manufacturers, reconstruction kernels, and slice thickness introduce “non-biological noise” that can overwhelm the true physiological signal. Feature harmonization as well as external validation are also essential to achieve generalizability required for clinical adoption.

To overcome reproducibility issues in CRC radiomics, recent high-quality studies have begun adopting standardized frameworks. For instance, the use of IBSI-compliant feature extraction and ICC thresholds for feature stability has been shown to mitigate scanner-related noise (98). Furthermore, the shift from internal cross-validation to independent external validation in multi-center cohorts ((99)) is now considered essential for demonstrating model generalizability and reducing the risk of overfitting.

#### 3.5.2 Liquid biopsy

Analysis of circulating tumor DNA (ctDNA), commonly referred to as liquid biopsy, is an emerging technique in the management of colorectal cancer across multiple stages, from locally advanced to metastatic disease (100).

A systematic review and meta-analysis by Wullaert et al. demonstrated a strong association between detectable ctDNA after treatment and adverse oncological outcomes in patients with colorectal liver metastases (CRLM) (101). This meta-analysis, which included nine studies, showed that patients with detectable ctDNA following surgery had a significantly higher risk of recurrence and shorter overall survival compared with those who were ctDNA-negative. A similarly strong association with recurrence was observed in patients with detectable ctDNA after completion of adjuvant therapy.

In a study by Wehrle et al., routine ctDNA monitoring in CRLM patients was shown to be logistically feasible (102). Their findings indicated that liver resection and/or transplantation can achieve clearance of detectable ctDNA, whereas failure to achieve clearance was predictive of increased relapse risk.

A subgroup analysis from the CIRCULATE-Japan GALAXY study by Kataoka et al. suggested that adjuvant chemotherapy provides significant clinical benefit to CRLM patients who are positive for molecular residual disease (MRD) after surgery In this MRD-positive cohort, 24-month disease-free survival (DFS) was significantly higher among patients who received adjuvant chemotherapy compared with those who did not. Conversely, no DFS benefit was observed in MRD-negative patients. MRD positivity was identified as the most significant prognostic factor for inferior DFS. Furthermore, recurrence patterns differed: MRD-positive patients were more likely to relapse in the liver, whereas MRD-negative patients more frequently recurred at extrahepatic sites such as the lung. Finally, results from the MIRACLE trial were recently reported, confirming the negative prognostic value of postoperative ctDNA and circulating tumor cell (CTC) detection following CRLM resection (103).

Different methodologies (tumor-informed vs. tumor-agnostic), different timing and risk of false negative results according to metastatic site and tumor shedding currently limit generalization of these results and prospective validation remains essential to effectively translate these tests into clinical practice.

## 4 Discussion

Modern management of oligometastatic CRC hinges on multidisciplinary integration of tumor biology, metastatic site, and patient-specific factors. Surgical metastasectomy remains the standard of care in properly selected patients for liver and lung metastases, but the landscape is evolving. Thermal ablation has demonstrated non-inferiority to surgery for small CRLM—with a superior safety profile—as suggested by the COLLISION and MAVERRIC trials, while SBRT offers a non-invasive option for anatomically challenging lesions or surgically unfit patients. Intra-arterial therapies further broaden the available armamentarium across curative and palliative settings.

The optimal integration of local and systemic therapy remains under investigation. Perioperative chemotherapy improves progression-free survival in resectable disease, though its overall survival benefit remains uncertain. In initially unresectable disease, intensive conversion regimens—often incorporating bevacizumab or anti-EGFR agents—can achieve curative-intent resection in a meaningful proportion of patients.

Emerging technologies are poised to refine patient selection and response monitoring. Molecular profiling could potentially help distinguishing true oligometastatic from aggressive polymetastatic phenotypes in the near future, while radiomics and ctDNA-based liquid biopsy hold promise for surveillance, residual disease detection, and treatment guidance—pending prospective validation. Notably, sex and gender differences in OMD-CRC outcomes remain unexplored and warrant future investigation.

Key challenges persist. Biological complexity exceeds current clinical and numerical disease definitions. Center expertise and volume critically influence outcomes, raising equity concerns around access to specialized care and underscoring the need for standardized training and referral networks. Optimal sequencing of local and systemic therapies—particularly with immunotherapy and targeted agents—requires further study. International registries and harmonized trial designs will be essential to bridging these gaps and advancing a truly personalized, biology-driven approach to OMD-CRC.

In conclusion, oligometastatic CRC management has become a dynamic and increasingly precise field. Integrating advances in local techniques, tumor biology, and systemic therapy offers growing prospects for prolonged, high-quality survival—with the ultimate goal of tailoring each patient’s treatment to maximize curative potential.
